# Employment Status and Self-Reported Unmet Healthcare Needs among South Korean Employees

**DOI:** 10.3390/ijerph16010009

**Published:** 2018-12-20

**Authors:** Rangkyoung Ha, Kyunghee Jung-Choi, Chang-Yup Kim

**Affiliations:** 1 Department of Occupational and Environmental Medicine, College of Medicine, Ewha Womans University, Seoul 07985, Korea; rang7452@gmail.com; 2 Department of Health Policy and Management, Graduate School of Public Health, Seoul National University, Seoul 08826, Korea

**Keywords:** employment status, precarious workers, self-reported unmet healthcare needs, barriers to healthcare utilization

## Abstract

We aimed to examine the association between employment status and self-reported unmet healthcare needs and to identify factors influencing self-reported unmet healthcare needs by employment status. Nationally representative data from the 2012 Korea National Health and Nutrition Examination Survey were used. Participants were classified by employment status as either permanent or precarious workers. Explanatory variables included sociodemographic, labor-related, and health-related factors. Multivariate logistic regression ascertained the association between employment status and self-reported unmet healthcare needs and explanatory factors associated with self-reporting of unmet healthcare needs. Precarious workers had a higher prevalence of self-reported unmet healthcare needs than permanent workers, with a statistically significant odds ratio (OR) (1.74; 95% confidence interval (CI), 1.19–2.54). Male precarious workers working >40 h per week were more likely to self-report unmet needs than male precarious workers working <40 h (OR, 3.90; 95% CI, 1.40–10.87). Female precarious workers with a lower household income were about twice as likely to self-report unmet needs. Working hours and household income were significantly influential factors determining self-reporting of unmet healthcare needs, especially among precarious workers. Policy interventions to improve access to healthcare for precarious workers are needed.

## 1. Introduction

Unmet healthcare needs can be defined as the perceived need for healthcare that is not received [1] or the difference between the health services considered necessary and the health services actually provided [2]. Previous studies of unmet healthcare needs have mainly focused on and assessed subjective unmet needs [3].

The factors preventing people from receiving healthcare have been examined. Females [1,4], younger people [4], people with chronic conditions [5], and people whose self-reported health status is less than “good” [6] have been shown to be more likely to self-report unmet healthcare needs. Empirical evidence indicates that socioeconomic factors, such as educational level, occupation, and income are associated with not only health status [7,8] but also healthcare utilization [9]. People of lower socioeconomic status generally have greater healthcare needs and more diverse health problems [1]. Several studies have provided significant evidence regarding income-related inequalities in unmet healthcare needs [10,11,12]. Immigrant status [13] and economic activity status [14,15] have been shown to affect unmet healthcare needs in Europe. Baert et al. [14] found unemployed people were more likely to have self-reported unmet healthcare needs owing to cost compared with full-time workers. Also, Hernández-Quevedo et al. [15] found unemployed or self-employed people were more likely to report unmet healthcare needs. Unmet healthcare needs in Europe were shown to have increased due to political circumstances and economic crises [16,17]. Reasons for unmet healthcare needs can be classified into three types: (1) availability, including long waiting times or unavailable services; (2) accessibility, including financial or transportation barriers; and (3) acceptability, including busyness or ignoring health problems [18].

Over the past three decades, neo-liberalism has been characterized by minimizing government interventions and labor market regulations and maximizing competition for the market, which has evolved globally. This has led to the acceleration of labor flexibility [19] and the emergence of precarious employment [20], which has made it easier for employers to hire and fire employees [21], thereby reinforcing the power positions of employers with respect to workers [22]. Employment conditions and jobs have been affected by flexible forms of employment in negative ways, such as instability, low wages, lack of benefits, and poor working conditions [23]. This international trend has been mirrored in Korea. After the financial crisis of 1997, the proportion of precarious workers increased due to the introduction of an act that protected dispatched workers, which was at the core of indirect employment and layoffs [24].

Although employment status is an important socioeconomic position indicator [25,26], as far as we know, there are no published studies from Asian countries concerning the association between self-reported unmet healthcare needs and employment status. We examined whether employment status is related to self-reporting of unmet healthcare needs. Our objectives were to determine if employment status is associated with self-reporting of unmet healthcare needs and to identify factors associated with self-reporting of unmet healthcare needs.

## 2. Methods

### 2.1. Data Sources and Study Subjects

This study analyzed data from the fifth Korea National Health and Nutrition Examination Survey (KNHANES V-3, 2012), which was conducted by the Korea Centers for Disease Control and Prevention (KCDC). This is an annual, nationwide, and representative cross-sectional survey, which contains around 10,000 individuals on a yearly basis. The KNHANES is conducted using a multi-stage, clustered probability design, and it consists of a health survey, physical examinations, and a nutrition survey [27]. These components collect information on socioeconomic status, healthcare utilization, health-related behaviors, quality of life, anthropometric measures, and dietary behaviors. This study employed the health survey, comprising demographics, education and occupation, healthcare utilization, activity limitations, and quality of life [28].

**Figure 1:**
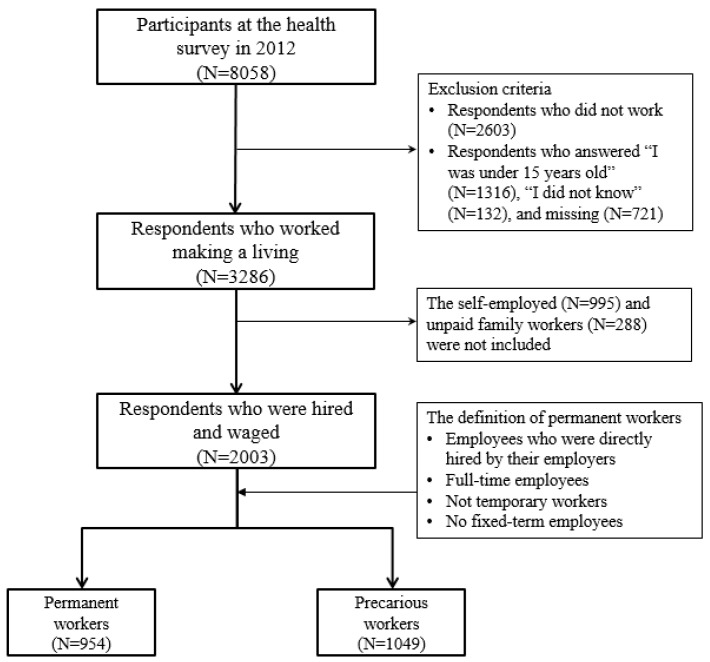
Selection of study subjects from the Korea National Health and Nutrition Examination Survey (KNHANES).

Among a total of 8058 participants in 2012, our analysis was limited to 3286 workers 15 years old or older. Self-employed and unpaid family workers were excluded. Our final analysis included 2003 participants: 954 permanent workers and 1049 precarious workers (Figure 1).

### 2.2. Study Variables

Participants were classified by employment status as either permanent or precarious workers. Permanent workers were defined as people who were hired by their employers directly, worked full-time, did not have a temporary job, and had no fixed-term employment contract [29]. Those who did not meet all four of these conditions were regarded as precarious workers.

The outcome variable—self-reported unmet healthcare needs—was annually measured using the question, “During the past 12 months, was there ever a time when you felt that you needed healthcare in hospital/clinics (excluding dental clinics), but you didn’t receive it?” Respondents who answered, “Yes, there was,” were considered to have self-reported unmet healthcare needs.

Explanatory variables were classified into three categories: sociodemographic factors, labor-related factors, and health-related factors. Sociodemographic factors consisted of sex, age, marital status, educational level, and equivalised household income. Marital status was classified as currently married, never married, or previously married. Educational level was divided into college or more, high school, and junior high or less. Equivalised household income, adjusted by the square root of household size [30], was classified into quartiles. Labor-related factors were: occupation, shift work, and average working hours per week. Occupations were categorized into three groups: professional/clerical, service/sales, and manual. Categories of average weekly working hours were: less than 40 h, 40 h, and more than 40 h. Shift work was based on whether the subjects worked during the daytime (6:00 am to 6:00 pm) or not and was divided into daytime and night shifts.

We used self-rated health status and limitations in activities of daily living as health-related factors. Self-reported health is helpful for measuring perceived population health status and predictors of healthcare needs [31]. Although there may be heterogeneity in reporting of health—varying by individual conceptions and expectations about health [32]—self-assessed health is feasible in large-scale surveys [31] and is extensively used for measuring socioeconomic inequality in health [33,34]. The question about self-rated health status was, “In general, how would you rate your health?” with possible answers being, “very good”, “good”, “moderate”, “poor”, or “very poor”. In the analysis, responses to the self-rated health status question were grouped as, “good (very good or good)”, “moderate”, and “bad (poor or very poor)”. Activity limitation was a dichotomous variable: limited versus not limited.

### 2.3. Data Analysis

Analyses for men and women were performed separately, considering the gendered labor market. Descriptive analyses were carried out for sociodemographics, labor-related factors, and health-related factors. General characteristics were analyzed to identify if there were any differences between permanent and precarious workers in terms of these characteristics. Additionally, the prevalence of self-reported unmet healthcare needs was calculated according to sex and employment status. Multivariate logistic regression analysis was used to examine the association between self-reported unmet healthcare needs and employment status and to ascertain what factors were associated with increased odds of self-reporting unmet healthcare needs. In the multivariate logistic regression model, the participants were stratified by sex and employment status. The associations and factors were presented as estimated odds ratios (ORs), with their 95% confidence intervals (CIs). We used SAS Survey Procedures (PROC SURVEYFREQ, PROC SURVEYLOGISTIC, SAS V9.4, SAS Institute, Cary, NC, USA) to account for the complex sampling design.

Availability of data and material: All data were publicly available on the website of the Korea National Health and Nutrition Examination Survey (https://knhanes.cdc.go.kr/knhanes/index.do).

### 2.4. Ethics Approval and Consent to Participate

The Institutional Review Board of Seoul National University, Seoul, Korea, approved this study (E1410/001-008 (2014.10.13)). Consent to participate was not required, as this study did not involve human participant interactions and all data were publicly available and de-identified.

## 3. Results

Table 1 shows the characteristics of study subjects according to sex and employment status. Compared with permanent workers, precarious workers were more likely to be women and tended to have less education and lower equivalised household incomes. Most of study subjects aged over 60 years were precarious workers. This pattern was similar when study subjects were divided by sex and by employment status. 

Male precarious workers were more likely to be manual workers, while a third of female precarious workers were service or sales workers. Among permanent workers, professional/clerical occupations were the most common in both genders. Regarding average working hours per week, male permanent and precarious workers and female permanent workers tended to work more than 40 h, while female precarious workers working less than 40 h were the most common.

Overall, 17% of employees reported unmet healthcare needs. The prevalence of self-reported unmet healthcare needs among permanent employees was 13.3%, and the prevalence among precarious employees was 20.7%. For male permanent and precarious workers, the prevalences of self-reported unmet healthcare needs were 10.0% and 18.1%, respectively. On the other hand, 19.5% of female permanent workers and 23.3% of female precarious workers reported unmet healthcare needs.

Table 2 illustrates the distribution of self-reported unmet healthcare needs prevalence according to the study subjects’ characteristics. Precarious workers with higher levels of education frequently reported unmet healthcare needs (20.5% among men and 25.0% among women). Female permanent workers with educational levels no higher than junior high school had a high prevalence of self-reported unmet healthcare needs. Especially among female precarious workers, participants from the first, second, and third household income quartiles reported similar rates of unmet healthcare needs. In terms of average working hours per week, the prevalence of unmet healthcare needs was the highest among employees who work more than 40 h regardless of employment status, in both genders. Also, the prevalence of self-reported unmet healthcare needs was high among permanent employees who reported limitations of daily living activities due to physical and mental disabilities.

The associations between self-reported unmet healthcare needs and employment status are described in Appendix Table A1. Precarious workers of both genders were more likely than permanent workers to report unmet healthcare needs (OR, 1.74; 95% CI, 1.19–2.54). This pattern was similar when study subjects were stratified by sex. Especially for men, statistically significant association was observed (OR, 2.16; 95% CI, 1.20–3.90).

We reanalyzed the data after dividing precarious workers into part-time workers and all other precarious workers, to concern the difference of working hours between two groups. The effect size of non–part-time precarious workers was higher than that of precarious workers (Appendix Table A1).

The factors associated with self-reported unmet healthcare needs are shown in Table 3. Working an average of more than 40 h a week was significantly associated with self-reporting of unmet healthcare needs among both permanent (OR, 1.98; 95% CI, 1.07–3.67) and precarious (OR, 2.68; 95% CI, 1.37–5.24) workers. When participants were stratified by sex and employment status, male permanent and precarious workers who worked more than 40 h were more likely to self-report unmet healthcare needs. Regarding equivalised household income among female precarious workers, participants within the third quartile, the second quartile, and the first quartile were more likely to self-report unmet healthcare needs than those within the fourth quartile. Furthermore, among participants with limitations of daily activities, the likelihood of reporting unmet healthcare needs increased in both male and female permanent workers.

The pattern of effect size was similar after adding the part-time employment subgrouping in the analysis of explanatory factors associated with self-reported unmet healthcare needs among precarious workers (Appendix Table A2).

## 4. Discussion

This study found that both male and female precarious workers had higher odds of self-reporting unmet healthcare needs than permanent workers. For men, the working hours was significantly associated with self-reporting unmet healthcare needs regardless of employment status. The effect size of working more than 40 h on self-reported unmet healthcare needs was higher among male precarious workers than among male permanent workers. Female precarious employees were more likely to lack access to healthcare services owing to financial constraints.

Overall, 17% of all employees in this study self-reported unmet healthcare needs. This is similar to reports from previous studies [6,35,36]. Song et al. [6] found that 16.2% of study participants, in an adult Korean population, self-reported unmet healthcare needs. Also, according to KCDC [36], the prevalence of self-reported unmet healthcare needs among Korean adults in 2012 was 16.7%. In 2016, an estimated 4.5% of the population aged 16 years and older in the 28 member states the European Union (EU) self-reported unmet healthcare needs, according to the EU statistics [37]. Ronksley et al. [5] found that 12.2% of their Canadian adult study sample reported unmet healthcare needs. Ayanian et al. [38] found that long- and short-term uninsured adults, aged 18 to 64 years, in the USA, were more likely than insured adults to report unmet healthcare needs (26.8%, 21.7%, and 8.2% respectively). Given the findings from the USA and given that health insurance is compulsory in South Korea, it could be said that the prevalence of self-reported unmet healthcare needs in this study was unexpectedly high. 

In our study, precariously employed study participants were 1.74 times more likely to self-report unmet healthcare needs than permanent employees, and working for more than 40 h per week was a major reason for this. Clinic and hospital hours for doctors in Korea are usually between 10:00 am and 6:00 pm, so these hours of operation might be considered constraints on receiving health services. The effect size of precarious workers who worked more than 40 h was larger than that of permanent workers who worked more than 40 h in both genders. A possible explanation could be that precarious employment is characterized by low job control at work [39,40], low wages, and high job insecurity [20,25]. These features could be related to a lack of schedule control at work, which makes it difficult to receive healthcare even when the perceived need arises.

There were economic reasons associated with self-reporting of unmet healthcare needs in women. Female precarious employees with lower incomes were more than twice as likely to self-report unmet healthcare needs because of costs. This might be because the wages of female precarious workers are lower than the wages of male precarious workers or female permanent workers. Even though women in precarious employment work the same amount of time or at the same occupations, there are wage disparities in Korean labor markets [41]. Also, although the health system in South Korea is based on compulsory health insurance and universal health coverage, there are not only co-payments for insured services but also out-of-pocket payments for uninsured services that can be barriers to receiving care [42]. Inpatients pay 20% of the cost for covered services, but cost-sharing of covered services ranges from 30% to 60% among outpatients [43]. According to Organisation for Economic Co-operation and Development (OECD) Health Statistics 2015 [44], 37% of the total health expenditure was from out-of-pocket payments in South Korea. This was nearly twice as high as in the OECD average (19%). Given these points, female precarious workers may face economic barriers to healthcare utilization.

Women in our study were more likely than men to self-report unmet healthcare needs. A possible explanation could be related to family care and housework [1,45,46]. Women are often engaged in both paid work and unpaid household care, so their double roles may lead to barriers to accessing healthcare. Indeed, a previous study identified that among dual-income couples, Korean women spent 6.5 times more hours on housework than Korean men [47]. There is still a social context of Confucian patriarchy in South Korea, so Korean women generally take on the larger proportion of responsibility for domestic work [48]. These expected gender roles lead to a higher risk of unmet healthcare needs for women.

Some limitations of this study should be pointed out. First, self-reporting of unmet healthcare needs relies on subjective responses, which are prone to recall bias, leading to the possibility of underestimation or overestimation. Additionally, it could be difficult to verify whether self-reported unmet healthcare needs were based on actual clinical needs or personal expectations, because unmet needs were investigated via a single question [49]. Despite these limitations, many studies have reported important findings using this variable of unmet healthcare needs from the KNHAES. Kim et al. [50] analyzed the data to compare with unmet healthcare needs in the EU and found the association between unmet healthcare needs and the share of out-of-pocket payments in Korea. Self-reported unmet healthcare needs were concentrated among lower income groups when Concentration Index (CI) was calculated [3], and education was an important factor associated with unmet healthcare needs [4].

Second, even though the KNHANES is a cross-sectional survey of a nationally representative sample, the number of study subjects representing each covariate was partially insufficient. This could have reduced the statistical power of this study.

Third, although we considered both self-rated health and activities of daily living, there are countless health status and determinants not investigated in our study. Those who have physical or mental disorders are more likely to report unmet healthcare needs. Thus, it would be important to consider medical history in investigating unmet healthcare needs.

Also, this study did not investigate job control at work or job security, which might explain the association between employment status and self-reported unmet healthcare needs. Further research is needed to examine these variables. Furthermore, this study did not consider business size as an explanatory variable, and one of the factors affecting self-reported unmet healthcare needs could be the sizes of the businesses where participants are employed. According to Article 16 of the Enforcement Decree of the Occupational Safety and Health Act in Korea, there is no need to appoint a health manager in a workplace ordinarily consisting of less than 50 workers. In such contexts, it might be difficult to receive care within the workplace, resulting in unmet healthcare needs among workers. 

Lastly, this study was unable to analyze longitudinal changes in self-reported unmet healthcare needs, as it was a cross-sectional study. Further research is needed to assess the trends and changes in unmet needs for healthcare over time.

Despite these limitations, the present study has several strengths. First, this study used data from a large study population that is nationally representative in Korea. Second, findings from this research complemented previous studies showing self-reported unmet healthcare needs among adults [18,51,52] by targeting employees. Third, previous studies have mainly focused on the economic reasons related to unmet healthcare needs in Korea [3,53], but this present analysis explored both economic and temporal aspects. Lastly, a strength of this study was its ability to identify the odds of self-reported unmet healthcare needs and factors associated with self-reported unmet healthcare needs stratified by sex and employment status.

## 5. Conclusions

This study identified that employment status was associated with self-reported unmet healthcare needs. The results of this study provide empirical evidence from Korea that precarious workers perceived more unmet healthcare needs, in contrast with permanent workers. Working hours and income were crucial in their influence of self-reported unmet healthcare needs, especially among precarious workers. Employment and working conditions, as well as healthcare system-level factors, should be considered in order to improve healthcare access. Furthermore, policy interventions to reduce inequities in healthcare access among precarious workers are needed.

## Figures and Tables

**Table 1:** Characteristics of study subjects by sex and employment status.

Variables	*N* (Weighted %)					
	Total (*N* = 2003)		Men (*N* = 998)		Women (*N* = 1005)	
	Permanent Workers	Precarious Workers	Permanent Workers	Precarious Workers	Permanent Workers	Precarious Workers
Total	954 (100.0)	1049 (100.0)	582 (100.0)	416 (100.0)	372 (100.0)	633 (100.0)
Sex						
Men	582 (65.6)	416 (48.6)				
Women	372 (34.4)	633 (51.4)				
Age (years)						
15–29	138 (19.8)	206 (29.7)	51 (14.1)	79 (30.0)	87 (30.6)	127 (29.4)
30–39	301 (32.1)	203 (21.1)	193 (35.2)	84 (23.9)	108 (26.0)	119 (18.4)
40–49	277 (30.2)	181 (19.5)	179 (31.2)	64 (18.0)	98 (28.4)	117 (21.0)
50–59	187 (15.3)	186 (15.0)	126 (16.6)	57 (12.6)	61 (12.8)	129 (17.2)
≥60	51 (2.6)	273 (14.7)	33 (2.9)	132 (15.4)	18 (2.2)	141 (14.1)
Marital status						
Currently married	718 (70.0)	679 (55.7)	490 (78.4)	284 (54.4)	228 (54.1)	395 (56.9)
Never married	195 (25.2)	255 (36.1)	81 (19.2)	114 (41.5)	114 (36.8)	141 (31.1)
Previously married	41 (4.7)	114 (8.2)	11 (2.5)	18 (4.1)	30 (9.1)	96 (12.0)
Education						
≥College	539 (53.7)	296 (29.9)	339 (54.6)	138 (35.1)	200 (52.1)	158 (25.0)
High school	336 (38.9)	400 (44.0)	204 (38.9)	160 (44.1)	132 (38.9)	240 (44.0)
≤Junior high school	79 (7.3)	353 (26.0)	39 (6.5)	118 (20.8)	40 (9.0)	235 (31.0)
Equivalised household income						
4th quartile	398 (37.3)	253 (23.2)	237 (35.3)	90 (21.2)	161 (41.2)	163 (25.2)
3rd quartile	325 (35.7)	313 (33.9)	194 (35.6)	148 (39.3)	131 (35.9)	165 (28.7)
2nd quartile	190 (22.5)	321 (31.5)	127 (24.5)	129 (30.7)	63 (18.7)	192 (32.3)
1st quartile	34 (4.5)	152 (11.4)	19 (4.7)	45 (8.8)	15 (4.1)	107 (13.9)
Occupation						
Professional/clerical	587 (57.0)	327 (32.5)	345 (53.2)	128 (32.9)	242 (64.4)	199 (32.2)
Service/sales	115 (12.5)	226 (24.3)	46 (9.0)	44 (14.9)	69 (19.2)	182 (33.2)
Manual	250 (30.5)	494 (43.2)	190 (37.9)	243 (52.3)	60 (16.4)	251 (34.7)
Shift work						
Daytime	803 (84.2)	792 (75.5)	491 (85.5)	303 (75.2)	312 (81.6)	489 (75.7)
Night shifts	147 (15.8)	245 (24.5)	88 (14.5)	108 (24.8)	59 (18.4)	137 (24.3)
Average working hours per week						
<40 h	119 (12.0)	531 (45.7)	53 (9.0)	130(27.9)	66 (17.8)	401 (62.6)
40 h	260 (24.4)	153 (15.4)	146 (22.1)	70 (18.2)	114 (28.8)	83 (12.6)
>40 h	575 (63.6)	365 (38.9)	383 (68.9)	216 (53.9)	192 (53.3)	149 (24.8)
Self-rated health status						
Good	355 (34.8)	343 (34.3)	232 (37.6)	148 (39.1)	123 (29.4)	195 (29.8)
Moderate	512 (55.3)	546 (51.7)	304 (54.0)	217 (50.4)	208 (57.8)	329 (53.0)
Bad	87 (9.9)	159 (14.0)	46 (8.4)	51 (10.5)	41 (12.8)	108 (17.2)
Activities of daily living						
Not limited	924 (97.0)	982 (93.8)	564 (96.8)	391 (94.5)	360 (97.4)	591 (93.1)
Limited	30 (3.0)	67 (6.2)	18 (3.2)	25 (5.5)	12 (2.6)	42 (6.9)
Self-reported unmet healthcare needs						
Yes	120 (13.3)	190 (20.7)	51 (10.0)	59 (18.1)	69 (19.5)	131 (23.3)
No	834 (86.7)	859 (79.3)	531 (90.0)	357 (81.9)	303 (80.5)	502 (76.7)

**Table 2:** The distribution of the prevalence of self-reported unmet healthcare needs of study subjects by sex and employment status.

Variables	*N* (Weighted %)			
	Men (*N* = 998)		Women (*N* = 1005)	
	Permanent Workers	Precarious Workers	Permanent Workers	Precarious Workers
Total	51 (10.0)	59 (18.1)	69 (19.5)	131 (23.3)
Age (years)				
15–29	7 (15.5)	13 (18.9)	20 (26.8)	36 (27.1)
30–39	17 (7.7)	15 (17.8)	16 (15.9)	28 (29.0)
40–49	13 (8.9)	13 (25.9)	15 (12.5)	10 (12.7)
50–59	10 (11.2)	7 (17.9)	14 (26.0)	29 (25.6)
≥60	4 (17.7)	11 (8.1)	4 (14.8)	28 (20.7)
Marital status				
Currently married	39 (9.1)	36 (15.5)	41 (19.6)	69 (21.7)
Never married	11 (13.3)	20 (20.4)	23 (21.4)	40 (26.8)
Previously married	1 (14.5)	3 (29.4)	5 (11.8)	22 (21.9)
Education				
≥College	31 (9.3)	24 (20.5)	35 (18.5)	32 (25.0)
High school	18 (11.7)	19 (19.2)	25 (19.9)	48 (22.1)
≤Junior high school	2 (6.4)	16 (11.7)	9 (23.9)	51 (23.5)
Equivalised household income				
4th quartile	23 (9.7)	15 (17.0)	29 (18.5)	21 (14.6)
3rd quartile	15 (10.7)	21 (20.2)	27 (21.6)	36 (26.0)
2nd quartile	10 (7.8)	18 (20.4)	9 (15.6)	46 (26.8)
1st quartile	2 (17.9)	5 (6.0)	4 (35.1)	27 (25.7)
Occupation				
Professional/clerical	29 (9.2)	17 (17.0)	46 (20.6)	47 (26.6)
Service/sales	5 (14.6)	9 (27.7)	16 (20.6)	35 (23.4)
Manual	17 (10.2)	33 (16.2)	7 (14.5)	49 (20.1)
Shift work				
Daytime	46 (10.4)	45 (19.1)	58 (20.1)	98 (23.3)
Night shifts	5 (8.3)	14 (16.0)	11 (17.4)	29 (20.2)
Average working hours per week				
<40 h	2 (1.6)	13 (14.9)	9 (11.0)	76 (20.7)
40 h	7 (5.1)	6 (9.1)	17 (15.2)	14 (19.0)
>40 h	42 (12.7)	40 (22.8)	43 (24.7)	41 (31.8)
Self-rated health				
Good	13 (6.3)	26 (24.1)	12 (11.7)	22 (15.9)
Moderate	34 (12.9)	22 (12.5)	42 (19.8)	70 (24.2)
Bad	4 (8.0)	11 (22.5)	15 (36.4)	39 (33.3)
Activities of daily living				
Not limited	44 (8.3)	54 (18.3)	61 (17.9)	117 (22.7)
Limited	7 (63.3)	5 (15.4)	8 (82.9)	14 (30.4)

**Table 3:** Factors associated with self-reported unmet healthcare needs by employment status and sex.

Variables	Odds Ratio (95% Confidence Interval)					
	Total		Men		Women	
	Permanent	Precarious	Permanent	Precarious	Permanent	Precarious
Sex						
Men	1	1				
Women	2.29 (1.27–4.13)	1.44 (0.90–2.29)				
Age	0.99 (0.96–1.02)	0.99 (0.97–1.01)	1.01 (0.97–1.06)	1.00 (0.97–1.03)	0.95 (0.90–1.01)	0.98 (0.96–1.01)
Marital status						
Married	1	1	1	1	1	1
Never married	1.31 (0.59–2.95)	1.18 (0.59–2.36)	2.76 (1.01–7.55)	1.11 (0.37–3.38)	0.52 (0.20–1.37)	1.17 (0.47–2.88)
Previously married	0.99 (0.23–4.23)	1.29 (0.55–3.05)	3.44 (0.36–32.69)	3.00 (0.83–10.78)	0.34 (0.08–1.46)	0.79 (0.33–1.93)
Education						
≥College	1	1	1	1	1	1
High school	1.23 (0.62–2.44)	0.90 (0.51–1.59)	1.09 (0.40–2.94)	0.96 (0.39–2.35)	1.22 (0.48–3.14)	0.99 (0.46–2.09)
≤Junior high school	1.81 (0.52–6.25)	0.98 (0.51–1.88)	0.75 (0.10–5.75)	0.72 (0.27–1.95)	5.53 (1.05–29.2)	1.45 (0.53–3.97)
Equivalised household income						
4th quartile	1	1	1	1	1	1
3rd quartile	1.05 (0.61–1.79)	1.78 (1.04–3.07)	0.80 (0.34–1.90)	1.20 (0.46–3.14)	1.40 (0.64–3.08)	2.45 (1.23–4.89)
2nd quartile	0.71 (0.35–1.47)	1.80 (0.97–3.32)	0.80 (0.28–2.35)	1.43 (0.49–4.15)	0.78 (0.27–2.28)	2.26 (1.18–4.31)
1st quartile	1.34 (0.47–3.83)	1.29 (0.60–2.77)	0.71 (0.17–2.98)	0.38 (0.10–1.46)	5.13 (0.88–29.83)	2.76 (1.00–7.61)
Occupation						
Professional/clerical	1	1	1	1	1	1
Service/sales	1.17 (0.60–2.28)	1.16 (0.64–2.12)	0.97 (0.31–3.10)	2.14 (0.71–6.48)	1.10 (0.44–2.79)	0.82 (0.40–1.70)
Manual	0.71 (0.33–1.54)	0.85 (0.51–1.43)	0.84 (0.28–2.50)	0.93 (0.40–2.13)	0.35 (0.10–1.27)	0.62 (0.31–1.27)
Shift work						
Daytime	1	1	1	1	1	1
Night shifts	0.66 (0.29–1.52)	0.65 (0.38–1.11)	0.61 (0.14–2.70)	0.56 (0.24–1.27)	0.71 (0.26–1.95)	0.61 (0.30–1.27)
Average working hours per week						
<40 h	1	1	1	1	1	1
40 h	0.61 (0.25–1.48)	1.57 (0.89–2.80)	0.42 (0.10–1.81)	2.16 (0.71–6.59)	0.56 (0.19–1.65)	1.42 (0.60–3.34)
>40 h	1.98 (1.07–3.67)	2.68 (1.37–5.24)	2.87 (1.13–7.29)	3.90 (1.40–10.87)	1.65 (0.74–3.71)	2.11 (0.81–5.51)
Self-rated health status						
Good	1	1	1	1	1	1
Moderate	1.84 (1.03–3.29)	0.86 (0.51–1.44)	1.89 (0.76–4.68)	0.49 (0.20–1.20)	2.07 (0.91–4.74)	1.56 (0.79–3.07)
Bad	1.50 (0.56–4.01)	1.65 (0.90–3.01)	0.49 (0.11–2.10)	0.97 (0.31–2.97)	3.15 (0.79–12.59)	2.52 (1.23–5.18)
Activities of daily living						
Not limited	1	1	1	1	1	1
Limited	15.42 (5.90–40.31)	1.06 (0.48–2.35)	22.69 (5.82–88.44)	0.85 (0.19–3.79)	14.02 (2.45–80.22)	1.29 (0.52–3.20)

## Appendix A

**Table A1:** Association between self-reported unmet healthcare needs and employment status by sex.

Variables	Odds Ratio (95% Confidence Interval)		
	Total *	Men **	Women **
Employment status1 ***			
Permanent workers	1	1	1
Precarious workers	1.74 (1.19–2.54)	2.16 (1.20–3.90)	1.28 (0.79–2.08)
Employment status2 ****			
Permanent workers	1	1	1
Precarious workers (excluding part-time workers)	1.87 (1.28–2.73)	2.36 (1.31–4.24)	1.31 (0.79–2.17)
Part-time workers	1.27 (0.72–2.23)	1.01 (0.30–3.44)	1.21 (0.62–2.36)

* Adjusted for sex, age, marital status, education, equivalised household income, occupation, shift work, average working hours per week, self-rated health status, and activities of daily living. ** Adjusted for age, marital status, education, equivalised household income, occupation, shift work, average working hours per week, self-rated health status, and activities of daily living. *** Employment status1 classified into permanent, and precarious workers. **** Employment status2 classified into permanent, precarious (excluding part-time), and part-time workers.

**Table A2:** Factors associated with self-reported unmet healthcare needs after adding part-time employment by sex among precarious workers.

Variables	Odds Ratio (95% Confidence Interval)		
	Total	Men	Women
Sex			
Men	1		
Women	1.52 (0.96–2.43)		
Age	0.99 (0.97–1.01)	0.99 (0.96–1.02)	0.98 (0.96–1.01)
Marital status			
Married	1	1	1
Never married	1.16 (0.58–2.30)	1.20 (0.41–3.46)	1.15 (0.47–2.83)
Previously married	1.26 (0.51–3.11)	3.59 (0.80–16.13)	0.77 (0.31–1.89)
Education			
≥College	1	1	1
High school	0.93 (0.53–1.64)	1.05 (0.43–2.59)	0.99 (0.47–2.11)
≤Junior high school	0.99 (0.52–1.90)	0.79 (0.29–2.11)	1.44 (0.52–3.96)
Equivalised household income			
4th quartile	1	1	1
3rd quartile	1.72 (1.00–2.97)	1.15 (0.43–3.06)	2.40 (1.19–4.84)
2nd quartile	1.79 (0.97–3.31)	1.40 (0.47–4.21)	2.26 (1.18–4.31)
1st quartile	1.24 (0.57–2.68)	0.35 (0.08–1.50)	2.71 (1.00–7.34)
Occupation			
Professional/clerical	1	1	1
Service/sales	1.23 (0.67–2.26)	2.03 (0.69–6.01)	0.85 (0.41–1.76)
Manual	0.89 (0.53–1.51)	0.98 (0.41–2.33)	0.64 (0.31–1.30)
Shift work			
Daytime	1	1	1
Night shifts	0.68 (0.4–1.16)	0.61 (0.25–1.47)	0.62 (0.30–1.29)
Average working hours per week			
<40 h	1	1	1
40 h	2.17 (1.15–4.09)	4.00 (1.15–13.99)	1.61 (0.60–4.35)
>40 h	2.76 (1.41–5.41)	4.28 (1.53–12.00)	2.13 (0.81–5.59)
Self-rated health status			
Good	1	1	1
Moderate	0.82 (0.49–1.38)	0.41 (0.16–1.04)	1.54 (0.78–3.05)
Bad	1.56 (0.86–2.83)	0.93 (0.30–2.84)	2.47 (1.21–5.04)
Activities of daily living			
Not limited	1	1	1
Limited	1.07 (0.48–2.37)	0.90 (0.20–4.10)	1.27 (0.51–3.20)
Part-time employment			
No	1	1	1
Yes	0.57 (0.35–0.94)	0.28 (0.08–0.91)	0.81 (0.42–1.55)
